# Human Oral Stem Cells, Biomaterials and Extracellular Vesicles: A Promising Tool in Bone Tissue Repair

**DOI:** 10.3390/ijms20204987

**Published:** 2019-10-09

**Authors:** Oriana Trubiani, Guya D. Marconi, Sante D. Pierdomenico, Adriano Piattelli, Francesca Diomede, Jacopo Pizzicannella

**Affiliations:** 1 Department of Medical, Oral and Biotechnological Sciences, University “G. d’Annunzio” Chieti-Pescara, 66100 Chieti, Italy; guya.marconi@unich.it (G.D.M.); sante.pierdomenico@unich.it (S.D.P.); adriano.piattelli@unich.it (A.P.); francesca.diomede@unich.it (F.D.); jacopo.pizzicannella@unich.it (J.P.); 2 ASL02 Lanciano-Vasto-Chieti, Ss. Annunziata Hospital, 66100 Chieti, Italy

**Keywords:** bone regeneration, biomaterials, extracellular vesicles, oral stem cells, regenerative medicine

## Abstract

Tissue engineering and/or regenerative medicine are fields of life science exploiting both engineering and biological fundamentals to originate new tissues and organs and to induce the regeneration of damaged or diseased tissues and organs. In particular, de novo bone tissue regeneration requires a mechanically competent osteo-conductive/inductive 3D biomaterial scaffold that guarantees the cell adhesion, proliferation, angiogenesis and differentiation into osteogenic lineage. Cellular components represent a key factor in tissue engineering and bone growth strategies take advantage from employment of mesenchymal stem cells (MSCs), an ideal cell source for tissue repair. Recently, the application of extracellular vesicles (EVs), isolated from stem cells, as cell-free therapy has emerged as a promising therapeutic strategy. This review aims at summarizing the recent and representative research on the bone tissue engineering field using a 3D scaffold enriched with human oral stem cells and their derivatives, EVs, as a promising therapeutic potential in the reconstructing of bone tissue defects.

## 1. Introduction

The development of the vertebrate skeleton was described at physiological and histological level at the end of the 19th century. The vertebrate skeleton develops from three distinct embryonic lineages: Neuronal crest cells contribute to the craniofacial skeleton, sclerotome cells from somites give rise to axial skeleton and lateral plate mesoderm forms the appendicular component. Bones provide a mechanical and protective function, and also represent the housing for blood-forming marrow and serve as a reservoir for mineral ions or as a site for the regulation of calcium ion homeostasis. The properties of bone tissue do not persist constant with age; in fact they change during a life, increasing and decreasing in some of the functional aspects [1,2].

Physiological bone remodeling is a highly coordinated process, responsible for bone resorption and formation, and is necessary to repair damaged bone and to maintain mineral homeostasis. Bone remodelling is a series of well-orchestrated biological events that are regulated by complex interactions between the various cell types found in bone, primarily osteoblasts, osteoclasts and osteocytes. In detail, osteoblasts, originated from mesenchymal stem cells, are the bone-forming cells, composed of 6% of the total bone resident cell population, and are located along the bone surface; instead, osteocytes composed of 95% bone cellular niche are the most abundant and long-living cells with a lifespan of decades. These cells are known for their crucial role in bone formation and resorption. Contrary to osteoblasts, osteocytes are easily identified in a bone section due to their dendritic shape and localization within small lacuna spaces in the hard mineralized bone matrix. Cell morphology varies between rounded and elongated shapes depending on the bone type [3]. In recent years, new approaches for de novo skeletal tissue formation have been developed in the tissue engineering and regenerative medicine fields.

In last decades, many researchers focused their attention on stem cells isolated from the human oral cavity due to their easy access, multipotent differentiation potential and high proliferation ability [4,5,6]. Oral stem cells originate from neural crests and represent a transient population of embryonic pluripotent stem cells that are induced at the lateral margins of the neural plate and migrate extensively to populate a variety of tissues [7].

The presence of a scaffold constituted by biocompatible materials, alone or associated with stem cells, represents a fundamental element in tissue engineering and/or regenerative medicine applications. In fact, a large variety of implant devices have increased the quality of life of millions of people. The advancement of technology and the development of innovative medicine lead to continuous increase of the diversity of biomaterials. Biomaterial is any substance or a combination of substances, natural or synthetic, that can be used independently as a part of a system to increase or replaces tissues and organs of the body. Recently, the development of 3D printing biomaterials, including poly (lactide) (3D-PLA), enriched with human mesenchymal stem cells (hMSCs) and/or their derivatives, such as extracellular vesicles (EVs), has been achieving promising results, in particular in bone regeneration in calvaria defects, in association with an enhanced vascularization offering a novel regulatory system in osteo-angiogenesis evolution. EVs, shed by almost all cell types, appear as a new promising tool in the biomedical field for the function in which they transport target molecules. EVs have a huge potential as a tool for cell-free therapy in bone tissue metabolism and regeneration. EVs can be used as a therapeutic agent by themselves or as delivery systems. Mesenchymal stem cells (MSCs) are one of the most promising sources of EVs, especially exosomes. Mainly, MSCs are easy to obtain from all human tissues and they have a high ex vivo expansion capacity compatible with immortalization without compromising their therapeutic efficacy. These two facts are essential to establish a scalable and long-term source of well-characterized EVs. In addition, their immunomodulatory effect gives them and their derived EVs important features in autologous and allogenic therapeutic applications [8].

This review aims at summarizing the main strategies reported to date on the function of biomaterials enriched with human oral stem cells and/or their derivatives such as extracellular vesicles (EVs) as promising tools to improve bone tissue regeneration and repair [9].

## 2. Morphological Features and Phenotypic Characterization of Human Oral Stem Cells

Stem cells from oral tissue are clonogenic cells with self-renewal and multi-lineage differentiation abilities for a variety of cell types including neural cells, adipocytes, endothelial cells and odontoblasts [10,11].

To date, six different human dental stem cells have been described in the literature: Human dental pulp stem cells (DPSCs), human exfoliated deciduous teeth stem cells (SHED), human periodontal ligament stem cells (PDLSCs), human apical papilla stem cells (APSCs), human dental follicle stem cells (DFSCs) and human gingival mesenchymal stem cells (GMSCs) [12,13].

Primary cultures of oral stem cells are colonies of bipolar fibroblastoid cells with oval nuclei containing two or three nucleoli. Comparisons amongst DPSCs, SHEDs, PDLSCs and BMSCs (bone marrow stromal cells) have demonstrated that DPSCs, SHEDs and PDLSCs maintain a higher growth potential in comparison to BMSCs [14]. Commonly, a limited used of stem cells could be related to the fact that they do not propagate in vitro for long periods. Recent studies, specifically focused on dental stem cells, have demonstrated their excellent stability and plasticity, without compromising their normal karyotype, even after 60 population doublings. It has also been established that the expansion period should not be longer than 20 to 40 population doublings [15,16]. Our research group developed a serum-free culture system for the in vitro expansion of oral stem cells in order to use them in clinical application [17].

Clinical applications of cell therapy represent a complex challenge due to the difficulty of operating under strict good manufacturing procedures. It is not only necessary to validate the safety of the proposed approach but also, first and foremost, its feasibility, without compromising the safety of the patients and its reproducibility for the clinical application [18,19].

Our study was focused on hPDLSCs and autologous and easily obtainable cell populations showing the essential features for human clinical use.

They are collected by scraping of the alveolar crest and/or the horizontal fibers of the periodontal ligament tissue without morbidity for patients. The xeno-free culture maintained the characteristic immunophenotype as well as the cells grown in mediums that contain animal sera, usually foetal bovine serum (FBS), and cells also maintained the multipotent ability and their ex vivo expansive potential. They also were capable of differentiating into adipogenic, osteogenic and chondrogenic lineages. The proliferation rate of hPDLSCs, expanded under animal-serum free conditions, was significantly increased when compared to the hPDLSCs cultured with standard a medium containing FBS. The morphological features were similar to the typical MSCs shape, showing a fibroblastoid morphology with evident nuclei and nucleoli.

Cell in vitro senescence during manipulation, considered a fundamental to the clinical use, is one other major point to be considered. The maintenance of low grade of senescence large-scale expansion of MSCs is very crucial for stem cell transplantation. The continuous passages of adult MSCs for a longer period may affect the embryonic stemness properties, including the proliferation and potential differentiation ability [20]. In our recent study, we demonstrated that MSCs, derived from oral tissues, are able to maintain the gene expression of embryonic markers and proteins involved in the proliferation at long-term passage cultures, till passage 15 [21].

In our cellular model, stem cells derived from oral tissues, in particular hPDLSCs, hDPSCs and hGMSCs, were considered; the expression of surface antigen markers related with the MSC features; the degree of cell proliferation rate remained unchanged when compared to stem cells at passages 2 and 15. The analyses of senescence marker expression have demonstrated the safety of transplanting long-term cultured MSCs for stem cell therapy [21].

In clinical trials for stem cell therapy, it is necessary and helpful to study the expression of embryonic stem cell markers; however, stem cells are required in large quantity to accomplish any clinical trials. Thus, multiple subcultures from the starting cells are needed to obtain the optimal cell number for transplantation; then a simple manipulation and various in vitro passages are needful. Nevertheless, the properties of stemness, including the proliferation and differentiation ability, as well as genetic modifications, over time, can be modulated in the prolonged culture of these dental and other adult MSCs [22,23].

Eleuterio et al. displayed that hDPSCs, hPDLSCs and hBMSCs share similar expression profiles on common cell surface antigens. DPSCs, PDLSCs and human BMSCs lack the expression of hematopoietic markers such as CD14, CD18, CD24, CD34 and CD45 and they expressed CD29/integrin beta-1 cell surface receptor, CD44, CD90, CD73, CD105 and CD150 cell surface glycoproteins, CD59 glycoprotein and CD166 transnmembrane glycoprotein [24,25,26].

**Figure 1:**
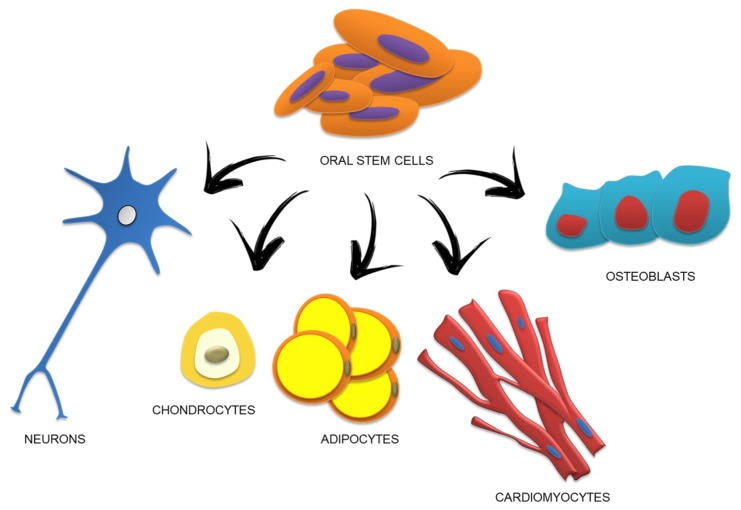
Oral stem cell potential differentiation ability.

Taking PDLSCs as an example, they showed a cell population able to differentiate into neural and mesodermal tissue [26], in particular into osteoblast/cementoblast-like cells, adipocytes and chondrogenic cells [27]. Recently, our research group reported that hPDLSCs expressed neural protein markers as Nestin and GAP-43 according to their neural crest origin. GAP-43 is expressed in glial cells of the nervous system and in the periodontal Ruffini endings, in which it has an important role in nerve regeneration/development processes [28,29]. Pizzicannella et al. demonstrated that PDLSCs could be induced to differentiate in endothelial cells and also in cardiomyocytes expressing sarcomeric actin and cardiac troponin T as cardiac cell markers [30]. Other authors reported the possibility of generating islet-like cells from PDLSCs expressing endoderm- and pancreas-related genes [31]. Moreover, the differentiation into retinal ganglion-like cells has been demonstrated in PDLSCs [32] (Figure 1).

The in vivo and in vitro experiments have demonstrated that the hPDLSCs are the master regulator of osteogenic differentiation. The hPDLSCs used in combination with different biomaterials were able to promote a bone regeneration process for the treatment of bone loss and ossification defects caused by senescence or accidental or surgery trauma. In fact, in our previous work we demonstrated that different types of biomaterials enriched by hPDLSCs and grafted in bone defects of mouse calvaria enhanced the bone regeneration process through the commitment and the induction of the differentiation of host cells [33].

Recently, Gingiva-derived MSCs (GMSCs), a novel type of multipotent MSCs showing potential self-renewing multipotent differentiation into several cell lineages and immunomodulatory capacities, has been described [34,35]. GMSCs attract an increasing consideration for their easy isolation, stable phenotype, normal karyotype, high proliferative capacity and telomerase activity after long-term culture [36]. GMSCs and PDLSCs are considered to be optimal candidates for tissue engineering and cell-based therapies [37]. For this reason the present review was focused on GMSC and PDLSC populations as stem cells used in bone skeletal segment reconstruction.

Nevertheless, oral MSC-based therapy is limited by the potential risk of unpredictable cell proliferation and differentiation. To avoid these risks, cell-free therapy appears as a promising strategy [38]. This cell-free therapy evidenced fewer side effects than MSC transplantation. Furthermore, MSC-derived EVs pass through capillaries more easily, whereas MSCs are too large to circulate through capillaries and a small proportion of them integrate in a perivascular niche.

## 3. Biogenesis and Characterization of EVs

EVs have huge potential as a tool for cell-free therapy in regenerative medicine. For instance, MSC-derived EVs are able to promote cell viability, proliferation, angiogenesis and immune responses. Exploiting the paracrine effects of stem and progenitor cells without having to administer living, replicating, potentially multipotent cell populations is an advantage in regard to safety, regulation and complexity. MSC-derived EVs are likely to obtain the osteogenic goal via modulating the cells and cytokines implicated in bone metabolism. In this review we summarized the function of EVs and MSCs in bone metabolism and regeneration. Recently, the development of biomaterials with the addition of stem cells gave promising results for the treatment of bone defects [39].

Nowadays, nano-sized vesicles, called extracellular vesicles (EVs), isolated from different cell sources represent a new tool for a regenerative and therapeutic approach to tissue regeneration applications [40]. For this reason, the International Society for Extracellular Vesicles (ISEV) has published position statements outlining criteria for defining these complex bioactive nanoparticles [41]. The latest research showed that EVs are associated with bone metabolism, bone healing and a potential capacity for mineralization [42]. Extracellular vesicles (EVs) are lipid bilayer-bound vesicles generated by cells with the property of being involved in intercellular communication. Released membrane vesicles from eukaryotic cells, as exosomes, microparticles, microvesicles and apoptotic bodies, can be retained as a dynamic extracellular vesicular compartment, strategic for their paracrine or autocrine biological effects in tissue metabolism [43]. In particular, EVs and their soluble secretory products, also called ‘secretome’, contain abundant proteins, nucleic acids, lipids and a pool of soluble cytokines; they seem to act as biomarkers in many cell functions [44]. Due to their size (50–1000 nm), EVs can circulate in the human body and in particular are present in most biological fluids. EVs are currently classified into three main types determined by their different biogenesis machinery. In brief, the first category is represented from exosomes, vesicles formed in multivesicular bodies (MVBs) and secreted by cells when MVBs fuse with the cytoplasmic membrane. The size of exosomes ranges between 50 and 150 nm, with density sizes from 1.15 to 1.19 g/mL. On the contrary, microvesicles (MVs) are generated through budding of the cell membrane. They show heterogeneous sizes ranging from 50 to 1000 nm with densities from 1.12 to 1.16 g/mL. A last category of EV subtypes consists of apoptotic bodies that are formed during the cell apoptotic process. Despite EVs’ promise of diagnostic and therapeutic applications, effective and pure isolation is still a problem that should be overcome. Specific protein markers for EVs have been planned so that isolation and verification could be easier. In fact, many markers in EVs have been evidenced, such as: Endosomal sorting complexes required for transport (ESCRT), system components (such as TSG101 and Alix) [45], tetraspanin proteins (such as CD9 and CD63) and some Rab small GTPase participating in the biogenesis of EVs (such as Rab27a, Rab27b and Rab35) [44]. Recently, EVs from GMSCs have been characterized through multiparametric analysis and they have been used in functionalizing the scaffold for bone tissue regeneration [9].

The DLS analysis of gingival EVs evidenced the presence of a heterogeneous population of EVs, spanning from 100 to 1200 nm. In particular, two main dimensional populations could be identified, the average diameter of the first population being 93 ± 24 nm and that of the second population being 1200 ± 400 nm with a ζ-potential of −10.7 ± 0.9 mV. Scanning microscopy analysis highlights the presence of a large number of globular EVs of different dimensions with a central depression, thus confirming previous reports on the shape of EVs as well as DLS data [46]. Moreover, human GMSC-derived EVs showed specific markers, such as: CD9, CD63, CD81, TSG101.

Functionalization of the EVs has not only enriched their functions, but also broadened their biomedical applications [47]. EVs can be functionalized to improve their performance using polyethylenimine (PEI), a synthetic polymer that covers EV surface. In our previous study, published by F. Diomede et al, the properties of collagen membrane-engineered scaffolds enriched with hPDLSCs and EVs or with hPDLSCs and PEI-EVs in bone regeneration through in vitro and in vivo evaluations were studied.

**Figure 2:**
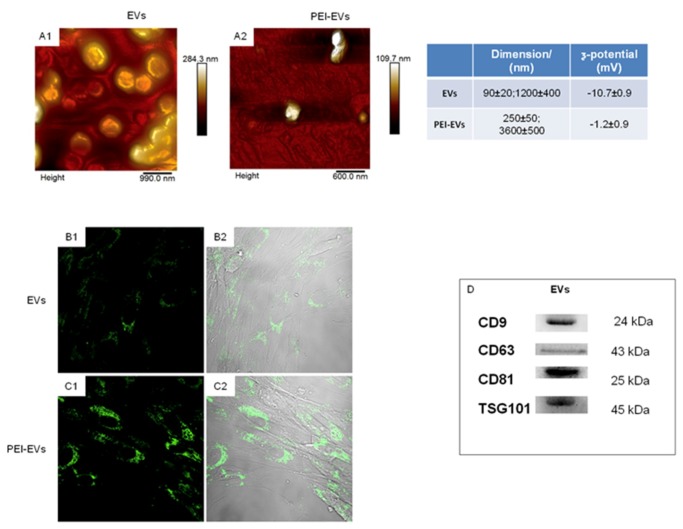
Extracellular vesicle (EV) and polyethyleneimine (PEI)-engineered EV (PEI-EV) characterization. (**A1**,**A2**) Atomic force microscopy pictures of EVs and PEI-EVs. (**Table**) Average size and ζ-potential of EVs and PEI-EVs. (**B1**,**B2**) Confocal laser scanning microscopy observations of fluorescent stained EVs cultured with GMSCs. (**C1**,**C2**) Confocal laser scanning microscopy observations of fluorescent stained PEI-EVs cultured with GMSCs. (**D**) Western blot showing the positivity for CD9, CD63, CD81 and TSG101. figure published in reference [9].

The study suggested that EVO enriched with hPDLSCs and PEI-EVs is capable of inducing bone regeneration. In particular, PEI-EVs played a key role in the activation of the osteogenic regenerative process. Indeed, the presence of PEI-EVs improved the mineralization process and induced an extensive vascular network, suggesting an osseointegration process. These data encourage a deep investigation of PEI-EVs in order to use them in bone tissue regeneration in combination with different types of scaffolds and SCs (Figure 2) [48].

## 4. Clinical Potential and Current Progress of Biomaterials and Human Oral Stem Cells in Bone Tissue Regeneration

The primary characteristics of bone tissue engineering involve the chemical composition and geometry of the scaffold designed to improve osteogenic differentiation; in fact, many studies have been carried out to better understand the role of biomaterials in the biochemistry and physiology of the bone tissue formation and vascularization. In particular, the use of scaffolds represents an integral part of bone tissue engineering, the composition and surface topography can have distinct effects on the local mediator production operated by cells, leading to downstream autocrine and paracrine regulation not only into neighboring cells, but also in cells that may be distant from the device [49,50]. Moreover, geometric characteristics can modulate the cell responses to local and systemic regulatory agents, in part through changes in the phenotypic expression and maturation state. All biomaterials, used for bone regeneration, should enhance bone growth directly in contact with the biomaterial surface from the surrounding bone (osteoconduction), but it should also be capable of inducing osteoinduction. An osteoinductive biomaterial should be able to recruit osteoprogenitor cells and transform an undifferentiated mesenchymal cell into an osteoblast [51]. Furthermore, It should allow colonization by the host blood vessels and be biocompatible and resorbable [52].

To understand the biological effects of the properties of biomaterials on biological functions, how these materials with manageable properties (e.g., compositional/degradable dynamics, mechanical properties, 2D topography and 3D geometry) can control cell behaviors (e.g., cell adhesion, spreading, proliferation, cell alignment and the differentiation or self-maintenance of stem cells) and tissue/organ functions needs to be taken into considerations [53,54].

These devices must have some fundamental features including biocompatibility, biodegradability, mechanical strength and matrix properties. Therefore, the novel design of three-dimensional printing biomaterials that could be used as bone substitutes considered to induce minimal or no immune response and promote implant/tissue interaction have been encouraged [55]. In bone tissue regeneration the ability to obtain an architectural reconstruction of efficient bone, during bone trauma, osteoporosis, arthritis, osteonecrosis and periodontitis, still remains an important goal. To enhance the bone repair process, the inflammatory response needs to be activated, while in the late bone remodeling the adaptive immune response is required.

Biomaterial remains a fundamental actor of this process, influencing the cell/tissue interactions, and its application still remains a promising strategy in bone tissue regeneration.

As extensively reported in the literature, several materials are currently used and widely studied for the construction of the scaffolds able to sustain the bone regenerative process [56].

In this review we focused our attention on two biomaterials: 3D-PLA and EVO membrane. In detail, the role of PLA in the regenerative medicine field is due to its biodegradability, biocompatibility, thermal plasticity and suitable mechanical properties [52]. The 3D-PLA was obtained from three-dimensional printing, which allows fabrication of complex scaffold designs with different interconnections, porosity and pore shapes that were previously difficult to build. In this review, we reported five innovative original designs of 3D-PLA scaffolds printed and their filament/pore sizes have been characterized.

The EVO membrane is a high-consistency dense collagen fiber derived from equine mesenchymal tissue. The major characteristics of this material are its maximum adaptability to hard and soft tissue, easy and secure suturability of nearby tissue, ample stability and sufficient protection of underlying grafts. In the tissue engineering field, the use of biomaterials enriched with mesenchymal stem cells is a new approach to promoting the reconstruction of bone critical defects [9].

The ability of cells to differentiate and adhere to biomaterials was affected by the mechanical properties of the scaffold, such as fiber width, porosity and matrix stiffness [57]. In particular, to induce cell osteogenic differentiation, harder scaffolds, composed by synthetic polymers, showed the best performances [56]. On the other hand, many studies demonstrated that a favorable regenerative microenvironment is created by ECM-based scaffolds, which lead to tissue remodeling and act as a template for the restoration of the bone tissue and other types of tissues [58]. The practice with ECM-based scaffolds displayed several advantages, such as low immunogenicity, differentiation and angiogenesis processes. Moreover, ECM scaffolds with a three-dimensional structure provide a mechanical support for the surrounding cells, constructing a favorable microenvironment that promotes cell adhesion and commitment during tissue regeneration, maintaining the original geometry and flexibility of the area regenerated [58].

Collagen-based materials are widely used during surgical and in postoperative surgical processes, and they can also have many other applications in different fields due to their primary role in the healing/regeneration process. Collagen has often been chosen in the tissue engineering field for its many advantages, e.g., its high biocompatibility, its low antigenicity with the main endogenous tissues and its biodegradability [59]. This protein is used in different forms such as sponges, injectable gels, films and membranes [60]. In particular, the collagen membranes, widely used in bone tissue engineering for their several positive effects, are able to promote the wound healing process, to reinforce a compromised tissue and to guide bone tissue regeneration in large skeletal defects [61]. The collagen-based materials can be considered an interface between natural and synthetic molecules, which are capable of stimulating the bone formation process in a damaged area [62].

Some molecules or ions were incorporated into bone scaffolds in order to provide a favorable microenvironment for MSC differentiation and also in order to enhance blood vessels and bone formation [63]. Collagen membranes can enrich with different bioactive molecules and stem cells in order to ameliorate and facilitate the healing process. For future clinical translation, it is mandatory to improve the efficacy of bone tissue-engineered scaffolds with cells and/or their secreted molecules, as well as the determination of the best biomaterial and optimal cell manipulation [64].

The most used synthetic and biodegradable scaffolds are PCL, PLGA and PLA scaffolds as well as their copolymers [65,66], especially PLA for its biodegradability, biocompatibility, thermal plasticity and suitable mechanical properties [67]. PLA can be used as a filament for three-dimensional printing, which allows the development of a complex scaffold designed with different interconnections, porosity and shape [68]. The absorbable polymers used in bone tissue engineering must ensure mechanical stability while degrading, thus keeping defect site stability during bone regeneration. Moreover, 3D-PLA printed scaffold biomaterial provided the best mechanical stability over time during their degradation [9].

Exceptionally intriguing are the data showing the upregulation of particular genes reported to be involved in osteoblast differentiation activated through TGF-β signaling, and these genes were found to be expressed at higher levels in 3DPLA + PEI-EVs + hGMSCs compared to hGMSCs [69].

**Figure 3:**
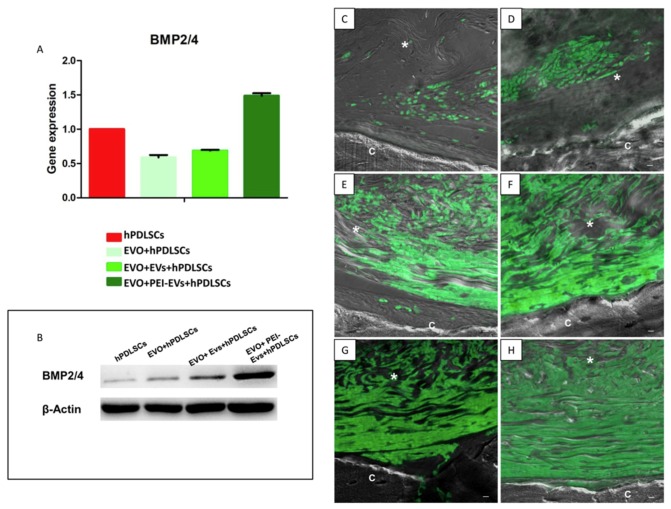
Collagen-based membrane in vivo functional evaluation. (**A**) Real time-PCR graph of BMP2/4 expression in different experimental groups after six weeks of in vitro culture (*n* = 3). (**B**) Western blot analysis of BMP2/4. Immunofluorescence staining of BMP2/4 showed the presence of the protein in semithin section samples obtained after six weeks of grafting in rat calvaria in (**C**) EVO, (**D**) EVO + hPDLSCs, (**E**) EVO + EVs, (**F**) EVO + EVs + hPDLSCs, (**G**) EVO + PEI-EVs and (**H**) EVO + PEI-EVs + hPDLSCs. Magnification: ×20; C, mouse calvarium; *, EVO. figure published in reference [48].

In vitro and in vivo analysis evidenced that the EVO membrane with a high-consistency dense collagen fiber derived from equine mesenchymal tissue enriched with hPDLSCs and PEI-EVs is able to promote a bone-regeneration process for the treatment of calvarium and ossification defects caused by accidental or surgery trauma. In particular, PEI-EVs had a significant role in the activation of the osteogenic process [48] (Figure 3).

In our previous studies, collagen-derived membranes were used. The evolution (EVO; Tecnoss Dental, Giaveno, Italy) three-dimensional membrane is a high-consistency dense collagen fiber derived from equine or bovine mesenchymal tissue. It was reported as a sterile dried membrane with smooth and microrough surfaces; its surface geometry has been observed through scanning electron microscopy. This membrane showed a high adaptability to hard and soft tissue, easy and secure suturability of nearby tissue, ample stability and sufficient protection of underlying grafts; these features allow the use of this material in different surgical procedures, making this technology easy to work in vivo or to manipulate in vivo [70].

Collagen-based materials are of extreme importance for tissue engineering and regenerative medicine due to their structure and high similarity to the main bone matrix components in vivo. Collagen membranes were reported to induce osteogenesis in situ [71].

Collagen is resistant to the proteolysis, although the peptide bonds in a triple helix are occluded from enzyme active sites, single-stranded regions are cleaved by matrix metalloproteinases (MMPs). High biocompatibility and intrinsic biodegradability by endogenous collagenases makes exogenous collagen ideal for use in biomedical applications [72]. Other than collagen-based membranes, several collagen-based biomaterials are used in clinical practice, such as collagenized porcine bone xenografts, biocompatible, bioabsorbable and also osteoconductive granule formulations both already tested in in vitro and in in vivo animal models [73]. Moreover, composites formed by porous sponge-like collagen, HA composites with ratios of 80:20 and 50:50, showed good biocompatibility and biomimetic properties and promoted adhesion and proliferation of MSCs, including hPDLSCs [74]. PDLSCs loaded on a biomimetic intrafibrillarly mineralized collagen scaffold exhibited excellent regeneration properties showing deposition of new bone with normal architecture and vascularization [75].

An EVO scaffold membrane, implanted in rat calvaria, did not present immunogenic effects; as previously demonstrated, after six weeks a porcine cortico-cancellous scaffold is able to induce the secretion of the extracellular matrix from numerous cells with fibroblast-like morphology [48].

Nowadays, the ability to increase osteoblast commitment using in vitro exosomes originating from mineralizing osteoblasts through the upregulation of β-catenin has been explored [76].

Exosome-functionalized β-TCP scaffold promotes bone repair and regeneration in a rat model of calvaria bone defects, opening a new strategy in the use of SC-secreted products as a potential therapeutic approach [77]. For instance, engineered polyethylenimine (PEI) EVs improved the mineralization process and induced an extensive vascular network in rat calvaria. PEI is a synthetic polymer with high cationic charge density due to the presence of protonatable amino groups [78]. PEI is generally used to deliver DNA molecules and small oligonucleotides. Furthermore, PEI showed in an in vitro model low toxicity and high biological activity after 24 h of treatment. This effect was likely due to PEI’s capacity to form non-convalent complexes with the DNA, which can be efficiently taken up by cells through endocytosis [78]. In our scientific project, EVs complexed with PEI are a strategic feature to induce bone regeneration, exhibiting a positive effect on cell morphology and gene transcription. These data support a new concept of personalized therapy, in which EVs and PEI-EVs may be collected by hPDLSCs and hGMSCs isolated from the patient, and which showed no immunorejection and infections once applied to the scaffold. Bone tissue engineering relies on the development of a vascular system designed for the delivery of oxygen and nutrients and also able to clear cell debris. This process, called angiogenesis, is regulated through a complex system of molecular signals as microRNAs (miRNAs), short noncoding RNAs composed of 20–22 nucleotides, which play an important role in the regulation of protein levels involved in the transduction of angiogenic signals [79,80]. In particular, miR-210 is involved in the inhibition of the expression of tumor suppressive genes and in the induction of cell proliferation and also plays a significant role in the regulation of angiogenesis correlated to VEGF expression [79]. PDLSCs grown in the three-dimensional inorganic bovine bone substitute showed an upregulation of miR-210 and VEGF. These results evidence that granules can be considered not only an adequate biocompatible three-dimensional biomaterial granules, but also an effective inductor of miR-210 and VEGF in the early steps of the bone-regeneration process. On the other hand, the 3D-COL enriched with hPDLSCs and PEI-EVs may promote bone regeneration of calvaria defects, and also presents an increase in vascularization evidenced through the upregulation of the vascular endothelial growth factor (VEGF) and VEGF receptor 2 (VEGFR2) [39].

The full treatment promotes the replacement of the damaged or non-functional tissues and the reconstruction of bone large skeletal defects caused by anomalies due to the effect of both soft and hard tissues by trauma and by bone recessions from tumors and cysts, or even by congenital disorders; the development of biomaterials functionalized with molecules or cell derivatives represents a socioeconomic need and a promising novel approach.

Biomaterials enriched with MSCs are capable of inducing bone regeneration. In particular, oral-derived stem cells, an alternative source of MSCs, represent a novel application in regenerative medicine, not only in dental restoration but also in other diseases. In fact, MSCs from oral tissues, presenting stem cell-associated cell surface markers, can be considered an interesting accessible autologous platform of stem cells. Moreover, dental MSCs, originating from neural crests, express embryonic stemness markers, which influence their multilineage differentiation potentials in vitro.

Oral-derived MSCs cultured in serum-free conditions do not interfere with their self-renewal and differentiation processes. They also present several advantages, such as large clinical-scale production of numerous competent stem cells without showing in vitro senescence for the passage from basic to translational research in transplantation, immune-therapy and regenerative medicine. In particular, the use of cell derivatives, as EVs or functionalized EVs, as PEI-EVs, played a key role in the activation of the osteogenic regenerative process, avoiding the direct use of stem cells with all their concerns. PEI-EVs improved the mineralization process and induced an extensive vascular network, suggesting an osseointegration process. These data encouraged a deep investigation of PEI-EV or MSC derivatives in order to use them in bone tissue regeneration in combination with different types of scaffolds and MSCs aimed at reaching a stem cell-free therapy approach.

Safety aspects must be highlighted from various perspectives (e.g., donor, recipient, product, manufacturing, clinical application). EVs will be considered biological medicinal products; it is anticipated that new rules explicitly regulating EV-based therapies are not needed. Existing European guidance on biological active substances covers the manufacturing and clinical evaluation of novel EV-based therapeutics. The demonstration of the safety and efficacy of novel drugs is a challenge for developers and clinical investigators.

## 5. Conclusions

The treatment for serious fractures and critical size bone defects requires the assistance of the tissue engineering field. For this reason, the identification of novel therapeutic strategies, which will lead to improved patient outcomes, are urgently needed. Hence, tissue engineering employs a combination of stem cells and their derivatives (EVs), biomaterials/scaffolds, to repair damaged bone and to enhance bone regrowth. This review aims at summarizing the recent and representative research in the bone tissue engineering field using different biomaterials such as 3D-PLA and EVO membrane alone and/or enriched with oral mesenchymal stem cells and their derivatives that can be used as a promising therapeutic approach in the reconstructing of bone tissue defects.
